# Breastfeeding, bonding, and olfaction: unlocking the potential of mother-infant odour exchange

**DOI:** 10.1016/j.ebiom.2025.106086

**Published:** 2025-12-20

**Authors:** S. Craig Roberts, Fabrice Damon, Karine Durand, Jan Havlíček, Dimitrios Kourtis, Ben Langford, Agnieszka Sorokowska, Piotr Sorokowski, Vivien Swanson, Jonathan Williams, Tatjana Arnoldi-Meadows, Dylan Brimaud, Daniela Dlouhá, Lucie Jelínková, Šárka Kaňková, Lenka Kapicová, Alice C. Poirier, Kateřina Roberts, Justus C. Sander, Dagmar Schwambergová, Michaela Silvestri, Nijing Wang, Emmanuel Simon, Benoist Schaal

**Affiliations:** a School of Psychology, University of Stirling, Stirling, UK; b Being Human Lab, University of Wroclaw, Wroclaw, Poland; c Development of Olfactory Communication and Cognition Laboratory, Université Bourgogne Europe, Institut Agro, CNRS, INR.AE, UMR CSGA, 21000 Dijon, France; d Department of Zoology, Faculty of Science, Charles University, Prague, Czech Republic; e UK Centre for Ecology & Hydrology, Penicuik, UK; f Institute of Psychology, University of Wroclaw, Wroclaw, Poland; g Max Planck Institute for Chemistry, Mainz, Germany; h Department of Philosophy and History of Science, Faculty of Science, Charles University, Prague, Czech Republic; i Obstetrics and Foetal Medicine, Université Bourgogne Europe, Dijon, France

**Keywords:** Olfactory communication, Smell, Attachment, Skin-to-skin, Breastfeeding, Perinatal care

## Abstract

Breastfeeding is crucial for infant survival, growth, and health, and it enhances maternal-infant bonding and well-being. However, breastfeeding rates typically fall below international targets, partly due to a high prevalence of latching difficulties, intermittent sucking, refusing the breast, or poor milk supply. Here, we propose that such uniquely human difficulties might be ameliorated by recognising, understanding, and facilitating the olfactory mechanisms that, in other mammals, regulate breastfeeding initiation and maternal–infant relationships in the first weeks of life. We briefly review evidence that odour mediates nipple-searching and suckling behaviour in other species, summarise the comparable evidence in humans, and outline pathways that could potentially reap hitherto unrealised benefits of olfactory communication between human mothers and neonates. We argue that enhanced awareness of such odour exchange could inform and enable changes in both policy and practice that might improve breastfeeding success and maternal-infant bonding, ultimately contributing to reduced infant mortality worldwide.

## Introduction

The benefits of breastfeeding are widespread and well-known. For infants, breastfeeding supports survival and long-term health outcomes, influencing immunity, metabolism, neurocognitive development, and mental well-being. For mothers, it plays a crucial role in bonding and recovery after childbirth while also reducing the risk of breast cancer. Recent estimates suggest that increasing breastfeeding rates could prevent 823,000 annual deaths in children under five and 20,000 annual deaths from breast cancer.^1^ Nonetheless, fewer than half of babies worldwide are breastfed within the first hour after birth or exclusively breastfed for the recommended six months,^2^ both key World Health Organization (WHO) and UNICEF targets.

Difficulties with breastfeeding initiation (e.g. latching, poor sucking, or refusing the breast) are pervasive even when mothers intend to breastfeed: a third^3^ to half^4^ have difficulties on the first day. Such difficulties may cause fatigue, pain, loss of confidence, and distress in mothers.^5^ For infants, they can pose clinical risks, lead to excessive weight loss,^3^^,^^4^ and deprive them of the earliest opportunity to ingest colostrum and non-pathogenic microbiota from the breast. Such consequences may be especially perilous in medically underdeveloped regions and may have been responsible for countless deaths in previous centuries.^6^

However, finding and latching successfully onto the breast is by no means a uniquely human challenge. By definition, it confronts neonates of every mammalian species. A universal solution—found in all female mammals studied to date—is the production of chemosensory signals from the mammary glands (or immediately adjacent sources). These odour messages take advantage of neonatal capabilities in olfaction, which often mature earlier than audition and vision.^7^^,^^8^

In this personal view paper, we propose that some of the difficulties with human breastfeeding might be ameliorated by recognising, understanding, and facilitating olfactory processes that underpin nipple-finding, latching, and sucking, especially in the critical first days of life. As in other social interactions in humans, these olfactory mechanisms have been widely underestimated and underappreciated. We advocate for a comprehensive reassessment of the importance of olfaction in mother–newborn interactions and describe how existing and developing knowledge of such processes could potentially be applied to inform systematic and transformational change in clinical practices around the mother and neonate that could have a significant positive impact on the initiation and subsequent maintenance of breastfeeding.

The need for such a reassessment is perhaps best illustrated by the fact that olfaction is not mentioned in the headline documents underpinning the WHO/UNICEF's Baby-Friendly Hospital Initiative (BFHI, revised in 2018), which aims to encourage the support of breastfeeding worldwide. Specifically, there is no explicit reference to odour or olfaction in its *Ten Steps to Successful Breastfeeding*, which outlines critical management procedures and clinical practices to promote optimal care for new mothers and infants, nor in the 56 pages of guidance provided for their implementation.^9^ However, as we will describe, a focused and explicit consideration of olfaction's role in the early stages of breastfeeding could have direct implications for each of the *Ten Steps*.

## The myth of poor human olfaction

The surprising omission of references to odour and olfaction in key policy and implementation documents may stem from the longstanding perception that olfaction is unimportant in humans. Research has now undermined this view to the point of it being termed ‘a 19th-century myth’.^10^ In fact, humans retain a neural architecture enabling us to outperform even ‘supersmeller species’ (dogs, mice) in detecting certain odours,^10^ and olfaction is increasingly recognised as playing a key role in our interactions with our environment.^11^ We use it in food selection and flavour perception, detecting environmental hazards and pathogens, and social communication.

Regarding the latter, natural (or even perfumed) body odours convey characteristics such as identity, age, gender, fertility, and affective state, mediating processes such as attraction, familiarity, and attachment.^12^ Yet, it has been repeatedly argued^7^^,^^8^^,^^13^^,^^14^ that these functions are overshadowed by the importance of olfaction in the critical maternal–infant relationship formed in the first days of life. As newborn infants are at their most vulnerable and otherwise sensorily limited, this is a unique moment when odours can communicate life-saving information.

## Across species, nipples convey a conspicuous ‘smellscape’

Indeed, every mammalian neonate urgently needs to find and latch onto a nipple or teat to receive nourishment and the full benefit of the protective properties of colostrum. Over evolutionary time, therefore, natural selection has apparently favoured the conspicuousness of the nipple.^7^ This conspicuousness need not be visual; indeed, the production of olfactory ‘beacons’ from the mammary glands appears to be a universal feature of female mammals.^7^

How do we know that the sense of smell is involved? In various species, behavioural evidence includes positive orientation of neonates towards swabs rubbed over the mammary area compared to swabs with saline solution^15^ and towards the odour of lactating over virgin females.^16^ Nipple search behaviour is hindered or eliminated by nostril obstruction or rendering neonates anosmic,^17^ and latencies to find the nipple and begin sucking are increased by washing nipples.^18^

**Fig. 1:**
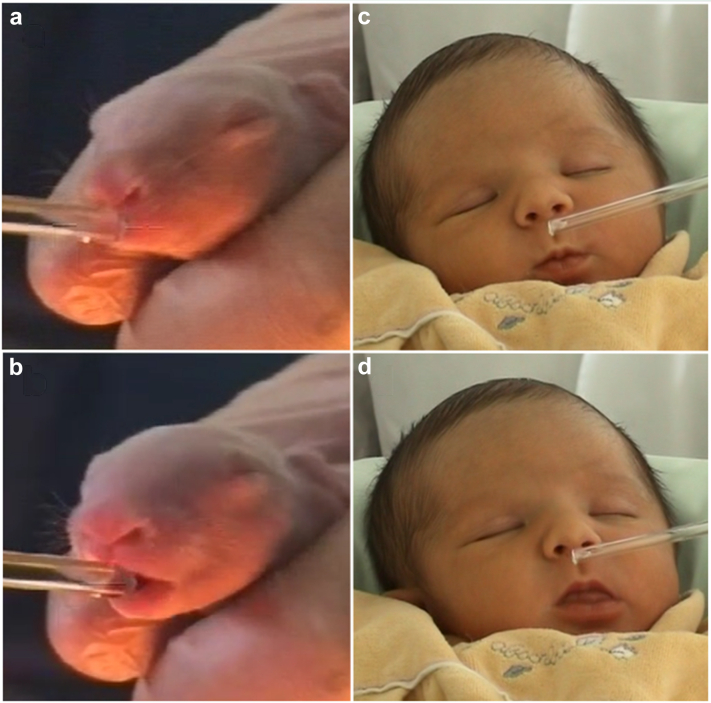
Responses to odours soon after birth. On the left, a rabbit pup (a) searches and (b) grasps a glass rod when it is coated in the rabbit mammary pheromone. On the right, a 3-day-old infant shows (c) lip pursing and (d) tongue protrusion in response to Montgomery's gland secretion (Photo credits: CSGA-CNRS).

However, we know most about odour-guided neonatal behaviour in the European rabbit, *Oryctolagus cuniculus*, in which selection pressure on nipple-finding is intense because pups are born ‘blind’ and mothers visit the nesting burrow only once daily, and then only for 3–5 min. This pressure apparently led to the evolution of the compound 2-methyl-2-butenal in rabbit milk, now known as the rabbit mammary pheromone,^19^ having met the criteria required to satisfy the formal definition of a pheromone.^13^ Such criteria include that it releases unambiguous and functional behaviour (in this case, searching, grasping, and sucking; Fig. 1), that the same behaviour is not elicited by other reference compounds or in other species, and that the responses should not (or only minimally) depend on previous exposure and learning. The pheromone is present at its highest concentrations immediately postpartum, declines during lactation, and is undetectable by weaning; this pattern is matched by pups' responsiveness, as neonates react more strongly to it than pre-weanlings.^20^ Pups that are naturally non-reactive to the mammary pheromone on day 1 die before weaning, demonstrating the consequences of natural selection in action.^21^ As in other species, latching is delayed by washing the abdominal fur of rabbit does or covering nipples with airtight rubber or film,^22^^,^^23^ and rendering pups anosmic leaves them unable to find the nipple.^23^

## Odour influences nipple-searching behaviour in infant humans

Although human mothers may bring their babies to the breast, human and rabbit infants share similar sensory abilities and vulnerabilities, and there is strong evidence that olfaction similarly plays a key role in breastfeeding initiation and success in humans.^8^ Infant rooting (bilateral head movements and oral searching for the nipple^24^) and mouthing behaviour^25^ is stimulated by elements of the maternal smellscape.^26^ Within minutes after birth, newborns positioned prone on the mother's abdomen can root and crawl independently towards the mother's breast,^27^^,^^28^ and breast odour is sufficient to elicit or enhance this movement.^29^, ^30^, ^31^ It also comforts infants, reducing crying^26^ and expressions of stress and pain.^32^ If the lactating breast is washed, they are less likely and take longer to find it.^29^ When the breast is covered with a scentless plastic film, infants are less attracted to it, exhibit fewer mouthing responses (Fig. 1), and are quicker to cry.^26^

It cannot be ruled out that learning of breast odour occurs in utero or immediately after birth, since babies can learn odours in amniotic fluid^33^ and within a few hours postpartum,^34^ and there are similarities in the chemical profiles of amniotic fluid and breast odour.^35^ Nonetheless, postnatal learning is not necessarily involved in the very earliest responses.^36^ Even infants who have been exclusively fed formula from birth orient their heads towards the breast odour of an unfamiliar, lactating woman more than the odour of their familiar formula^24^^,^^37^ and the breast odour of a non-lactating woman^38^; they also suck harder and more frequently when a mother's milk odour is presented under their noses when feeding compared with the odour of formula or water.^39^

## Sources of breast odour active components

As in rabbits, milk is a likely source of the active chemical compounds responsible for newborn attraction to human breast odour. At least 63 volatile compounds in breastmilk are detectable by the human nose.^40^ However, different volatiles are expressed in lacteal secretion at different times (this is likely a general feature of all mammals^41^). When compounds were compared across three lactational stages (colostrum, transitional milk, mature milk), some occurred at relatively higher abundance in colostrum^42^ and might, therefore, be critical in facilitating nipple-finding and breastfeeding initiation. Indeed, behavioural experiments indicate that colostrum is especially attractive to 2-day-old newborns compared to mature milk.^43^

Another potential source is skin glands surrounding the nipple, especially the Montgomery's glands (MGs, also known as areolar glands). Histological evidence shows these small protuberances combine both miniature mammary acini and sebaceous glands, the latter being involved in scent communication across taxa.^7^ Secretory MGs are unevenly distributed on the areola, being more common in the upper lateral quarter on each breast; such a distribution is indicative of communicatory function, as these quarters are those to which the feeding infant's nose is usually directed.^44^ The number of MGs on the breast of individual women varies, with greater numbers predicting faster re-gain of initial infant body mass, earlier onset of lactation, faster latching onto the nipple, and higher sucking intensity.^44^ Furthermore, the odour of MG secretion from an unfamiliar mother stimulates stronger oro-cephalic responses (lip-pursing, tongue protrusion; Fig. 1) in 3-day-old infants than an unfamiliar mother's milk, cow milk, or formula.^45^

## Odours influence maternal-infant bonding

So far, we have described maternal chemical signals (or chemosignals) that have evolved to communicate vital information to newborns; however, infant-produced odours may also alter maternal behaviour. In sheep, for example, ewes deprived of olfactory (but not auditory) cues from lambs show reduced care behaviour.^46^ In rats, production of the compound dodecyl propionate by the pup's preputial gland stimulates anogenital licking by the mother, which is critical for pup survival through stimulating excretory reflexes.^47^

In humans, infant odours appear to play a significant role in maternal bonding. The smell of a newborn baby is salient and attractive to post-parturient women^48^ and activates reward-related neural substrates,^49^ especially the odour of their own child.^50^ Mothers rapidly learn to distinguish their baby's smell^31^; this ability, and how much they express liking their infant's odour, predicts subjective ratings of maternal bonding^48^ and investment.^51^

Beyond their role in bonding, one can also speculate that the same evolutionary selection pressures that shape breast signals targeted at infants could lead to infant-produced chemosignals that communicate hunger or emotional state to caregivers. In rats, the odour of hungry individuals is known to elicit caring behaviours.^52^ In adult humans, hunger or satiety can be discriminated by smell,^53^ as can emotional states (e.g. fear, stress).^54^ Such findings raise the possibility that infant odours could communicate hunger or distress, acting as a signal of need, analogous to an ‘olfactory cry’, perhaps in advance of an auditory one. Even small uplifts in maternal attentiveness arising from such a signal could have life-saving effects, perhaps especially at night when vision is ineffective. Such uplifts might occur subconsciously, as subthreshold odours can affect behaviour in other contexts.^55^ We can even hypothesise that increasing expression of such a chemosignal as infants become hungry could trigger the mother's physiological pathways that lead to milk letdown and/or upregulation in the emission of the volatile compounds comprising the breast odour signal.

## Harnessing the benefits of odour communication in perinatal care

**Fig. 2:**
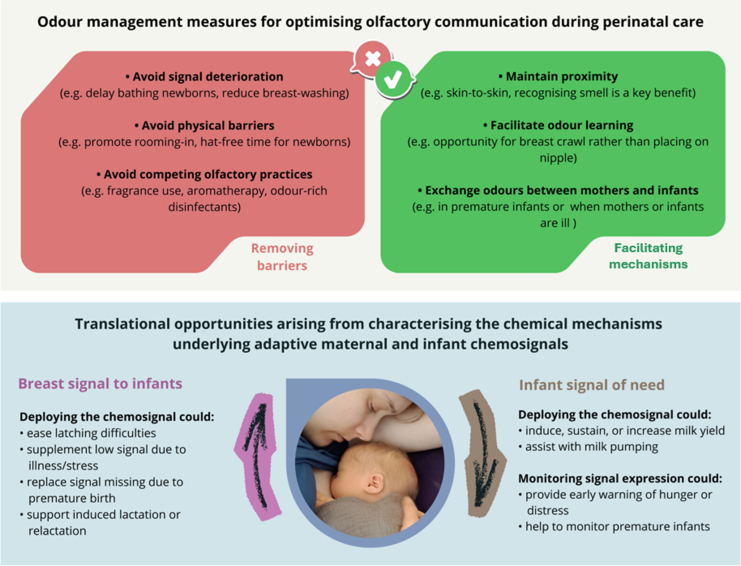
Opportunities for integrating understanding and awareness of maternal-infant olfactory communication in clinical practice. *Above*: Odour management measures in perinatal care can address barriers to odour communication or act to facilitate them. *Below*: Identifying the specific chemical compounds responsible for eliciting adaptive behaviours, including the chemosignal that attracts newborns to the nipple and a (hypothesised) signal of need/distress from infants, could lead to the development of isolated or synthetic chemosignals with defined practical applications.

There are several possible pathways by which emerging insights into maternal-infant olfactory communication in humans and other mammals could be harnessed to boost the success of practical initiatives, such as those aimed at increasing breastfeeding rates (Fig. 2). More research is needed, but the existing evidence is already sufficient to suggest that we should aim to boost practices that favour olfactory communication to establish and maintain breastfeeding and discourage those that hinder it. Further translational opportunities await the discoveries of the precise chemical mechanisms involved.

### Facilitating communicative mechanisms

The most obvious approach to boosting olfactory communication is maintaining mother-infant proximity in the earliest moments of postnatal interaction. Thus, providing rooming-in and skin-to-skin contact (hereafter, SSC)—both incorporated into the WHO/UNICEF's *Ten Steps*—are essential to enable communication in hospital settings (rooming-in does not apply when mothers give birth at home, as is increasingly common in some countries). Although the prevalence of SSC within the first hour of life varies hugely across the world, research demonstrates its well-known and varied benefits.^56^ In infants, for example, it is associated with reduced stress and crying, and increased rates of breastfeeding initiation and exclusive breastfeeding. For mothers, SSC is associated with reduced stress and increased breastfeeding self-efficacy, as well as the release of oxytocin, which enhances parental bonding and care behaviours, and boosts lactation itself.

Even in clinical settings that value SSC, the sensory mechanisms underpinning SSC benefits are often either not explicitly considered or assumed to be tactile.^57^ However, olfaction is likely to be equally or even more involved.^8^^,^^58^^,^^59^ At least four of Widström's nine critical behavioural phases^28^ during SSC in the first hour involve smell (stages 4 and 6–8: activity, crawling towards the breasts, familiarisation with the areola/nipple, and initiation of suckling).^28^^,^^60^ Appreciating that the main benefit of SSC might come via olfaction—at least as much as touch or vision, and perhaps interacting with them multimodally—is essential to shaping clinical practices that facilitate it.

Furthermore, there is evidence to suggest that providing newborns with the opportunity to use their sense of smell in rooting and crawling to the breast is an important factor influencing breastfeeding outcomes. Klaus^61^ noted that an infant who is permitted to crawl unaided to the breast “opens his mouth widely and, after several attempts, makes a perfect placement on the areola of the nipple”, while in those placed directly between the breasts “the infant reaches the target just as effectively, but with a different pattern of behaviour. Instead of beginning to suckle, many infants just lick the nipple and their hands” (p.1244). Righard and Alade^27^ reported that infants allowed to crawl unaided were more likely to begin breastfeeding and less likely to suck inefficiently or with a superficial latch, compared with those placed directly at the breast and whose mothers usually manipulated the nipple into the infant's mouth. A randomised controlled trial (RCT) recently showed that the crawl (facilitated by cheek-to-cheek contact and smearing the breast with expressed milk to enhance odour cues) led to reduced time to breastfeeding initiation and improved latching compared to SSC alone.^62^ While more studies are desirable, such findings suggest that active rooting and crawling provide opportunities for learning the mother's breast and body odour and how to respond appropriately. It might even form a critical sensitive stage^59^ (similar to that suggested for maternal bonding^63^^,^^64^), since subsequent recognition of maternal odour is improved in infants provided with early SSC^65^ and neural activation in response to the odour of colostrum is higher in the first 24 h than over subsequent days.^66^ Thus, the direct placement of a passive newborn on the breast may be (at first sight, counterintuitively) less beneficial, and have long-lasting effects on infants, compared to providing them with a reasonable and appropriately timed opportunity to actively and independently find the breast, even if this means they take longer to initiate feeding.

Beyond SSC, further research is also needed on interventions to enhance communication, particularly where direct communication is not possible. To date, most work has focused on premature births, where mothers and newborns are often physically separated. Welch et al.^67^ developed a ‘family nurture intervention’ (FNI) to counteract the adverse effects of separation in preterm families; among other intervention subcomponents, this included a protocol involving reciprocal exchange between mothers and infants of cotton cloths infused with body odour (worn in the bra or placed under/on the newborn's head). The FNI had several positive outcomes in terms of mother-infant engagement (touch, gaze),^68^ neurodevelopmental functioning (including cognition, language, attention, and social functioning up to 18 months, and theory-of-mind ability at age 4), and reduced rates of post-discharge anxiety and depression.^69^ Odour exchange is an intuitive approach, and parents readily grasp its potential benefits: sometimes even parents in the standard care group wanted to use it,^70^ and parents in one trial were more likely to engage with it than SSC, reporting that it enhanced feelings of attachment with the baby.^71^ However, it should be noted that the FNI is multimodal, and more work is needed to isolate effects of scent exchange from other subcomponents (e.g. soothing voice, touch, SSC).

More separable approaches include the use of maternal odour to facilitate feeding or relieve pain. One meta-analysis of six RCTs^72^ indicated that odour stimulation with breastmilk reduces the transition time from tube to oral feeding; in these studies, the preterm infant's own mother's breastmilk was applied to gauze pads held 2–5 cm from the infant's nose while tube-feeding. However, another meta-analysis of eight studies found no significant benefit,^73^ so further research is required on this issue. One study suggested a reduced likelihood of neurosensory impairment in premature infants exposed to the smell and taste of milk before gastric tube feeds.^74^ Finally, a meta-analysis of eight RCTs measuring pain during clinical interventions (e.g. venipuncture, heel-prick) concluded that, compared to no-odour controls, infants exposed to maternal odour before or during the intervention have lower pain scores and cry less.^75^ Odour presentation likely reduces pain either by influencing affect (such as diminishing fearfulness^76^) or by distraction.^77^ Overall, these studies demonstrate that odour exchange interventions could be effective in achieving multiple desirable outcomes for mothers and infants in clinical care.

### Removing barriers to odour communication

Clinical care practices that reduce the potential for effective odour transmission between mothers and infants should, as far as possible, be avoided. Perhaps the most obvious example is bathing newborns. Although the WHO recommends delaying the first bath for at least 24 h, bathing within 10 min of birth remains common in many countries.^78^ Not only will this interfere with SSC and autonomous breastfeeding initiation by the infant if bathing happens before the first breastfeed,^61^ it also disrupts maternal reception of infant odours during the same period. Similarly (but less often discussed), reducing the frequency of breast-washing in the first days may ensure that critical olfactory cues are maximally available for reception by infants.^29^

Physical barriers have been reduced since the broad adoption of the practice of rooming-in. However, the WHO's recommendation that babies have more layers of clothing than adults, and to keep the head covered with a hat, maintains a remaining physical barrier for the reception of infant odour by mothers; adjusting this guidance might be appropriate where thermoregulatory conditions and cultural traditions permit.

Furthermore, the introduction of olfactory interference in the birthing and early postnatal environment should be avoided. Competing olfactory practices might include the use of odour-rich antiseptic solutions,^79^ the hand odours of practitioners touching the mother's breasts,^60^ and perfume use by those around the mother. Odour-neutral disinfectants should likely be used where birthing and first breastfeeding episodes will occur, and it should be recognised that aromatherapy practices for pain relief in the second or third stages of labour^80^ could produce unwelcome olfactory interference.

Recommendations concerning mothers’ skincare or cosmetic use may be more nuanced. Ointments for sore or cracked nipples should ideally be unscented, and anointing breasts with scented balms or perfumes should likely be avoided, at least during the first days postpartum. However, artificial scents that mothers wear may be rapidly learnt by infants^81^ and appear to provide additional social cues (rather than superseding the natural breast chemosignal^82^). What may be most important, then, is consistency of product use: habitually changing products to suit social context or mood will likely become confusing for infants.

### Characterising the chemical mechanisms

Further research is required to characterise the volatile compound(s) produced by the lactating breast to which human newborns react, which would be a strong candidate for a mammary pheromone, similar to that in rabbits.^19^ Whether humans communicate using pheromones has been identified as one of science's outstanding questions,^83^ and there is consensus that a breast odour signal to infants is the likeliest candidate.^13^

However, identifying a human breast chemosignal may be more challenging than in rabbits. There are no guarantees that the active signal is comprised of a single compound, as in the rabbit: it may involve two or more compounds, perhaps occurring in specific ratios (as in some other social signals^84^). It may also be relatively transient, being expressed only shortly after birth or in bursts when infants are hungry. We also face additional practical and ethical constraints in research involving human newborns and parents. Thus, while a convincing characterisation of a human chemosignal would depend on a similar systematic, stepwise approach^13^ as used in the rabbit research, achieving it may require innovative methodological solutions.

One such advance could involve replacing the use of gas chromatography–mass spectrometry (currently used in most studies on mammalian olfactory communication and limited to providing single-timepoint analyses of odour samples) with continuous, real-time measurement of odour changes in undisturbed individuals, using techniques drawn from environmental and atmospheric chemistry.^85^ This approach would enable the identification of compounds that are expressed at higher levels in specific relevant contexts and for short periods, such as when babies become distressed or hungry. We must also identify the appropriate methods to optimally evaluate responses to odours, which may involve working with sleeping newborns because psychophysiological measures are challenging in awake infants and stimulus-specific autonomic responses appear more reliable during sleep.^45^ Finally, a conclusive identification is likely to depend on confirmation that the chemosignal is produced and responded to in the same way across genetically and culturally diverse human populations.

Although these challenges are significant, success could provide novel approaches to address breastfeeding difficulties directly. For example, the successful identification of a mammary pheromone could enable the development of a synthetic version that is attractive to newborns; this could be applied to the breasts of mothers whose babies refuse to feed or have latching difficulties, or to boost maternal self-confidence in the first few days (especially in primiparas). It might also be used to supplement the odour of mothers who are ill, who give birth prematurely or by caesarean section, who are exposed to stressful situations that interfere with lactation,^86^ or who attempt induced lactation or relactation (e.g. adoptive mothers, same-sex couples).^87^

Similarly, being able to detect an infant-derived chemosignal of need might provide early warnings of hunger or distress that could, for example, be used to remotely monitor the arousal of premature babies. A synthetic version of this chemosignal could be deployed to help induce, sustain, or increase lactation and milk yield, or to assist with breast-pumping to obtain sufficient milk for a separated (e.g. preterm or ill) infant.^88^

While these varied possibilities are intriguing, we would be remiss if we did not also flag potential concerns regarding undesirable applications and consequences. Foremost among these is that the development of a synthetic breast attractant should never undermine efforts to support routine breastfeeding initiation and maintenance. All aspects related to potential commercialisation should be subject to rigorous ethical scrutiny before any implementation.

## Implications for international breastfeeding policy

In our view, the starkest representation of a persistent underappreciation of maternal-infant odour communication is in policy-making around breastfeeding. As described above, this underappreciation is perhaps best illustrated by the fact that there is no explicit reference to olfaction in the headline documents underpinning the WHO/UNICEF's BFHI, neither in the *Ten Steps to Successful Breastfeeding* nor in the implementation guidance.^9^

We therefore suggest that a focused analysis of policy documentation from an olfactory perspective is urgently needed. To pick one notable example, it should now be clear why we would recommend rewording of information in the section on Step 4 (immediate postnatal care) in the BFHI Implementation Guidance, where it states: ‘Immediate and uninterrupted skin-to-skin contact facilitates the newborn's natural rooting reflex that helps to imprint the behaviour of *looking for* the breast’ (p.17, our italics). Such wording appears to be symptomatic of an apparent underestimation or even disregard for the important role played by the sense of smell.

**Fig. 3:**
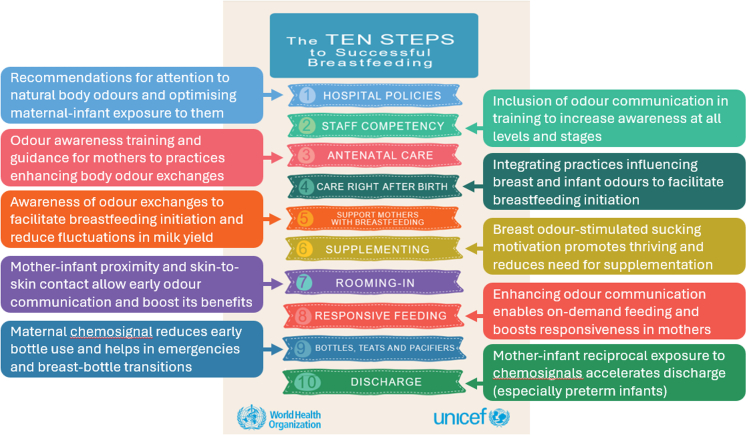
Ten ways that enhanced understanding and awareness of mother-infant odour communication can already—or potentially could—contribute to the WHO/UNICEF's *Ten Steps to Successful Breastfeeding*.

Indeed, we believe that a thorough and explicit consideration of the role of odour in the early stages of breastfeeding could produce evidence and insights that eventually lead to benefits on each of the *Ten Steps* (see Fig. 3). First (Step 1), recommendations for attention towards natural body odours and optimisation of exposure to these in mother-infant dyads, incorporating at least some of the points we have reviewed here, could be integrated into hospital policies. Knowledge of odour communication could be incorporated into the training of healthcare professionals to enhance staff awareness and competency, and odour awareness training and guidance could be integrated into antenatal care for prospective parents (Steps 2 and 3). Such improved awareness would be expected to inform practices involving mammary and infant odour substrates, facilitating breastfeeding initiation, colostrum intake, and reducing early fluctuations in milk yield (Steps 4 and 5). Cumulatively, these steps could be expected to reduce the need for supplementary feeding (Step 6) and improve responsive feeding (Step 8), while awareness of the importance of odour reinforces the widely (but still not universally) recognised benefits of rooming-in, while also clarifying why rooming-in and SSC are so critical (Step 7). Furthermore, such measures should reduce the need for bottle-feeding, while maternal odour can be used, if necessary, to facilitate breast–bottle transitions (Step 9). Finally, reciprocal exposure to maternal and neonatal odours may reduce the time to discharge from hospital (Step 10), especially in premature infants (e.g. if further studies confirm that exposure accelerates the transition from tube-feeding to autonomous breastfeeding).

## Conclusions and future directions

In humans, as in other mammals, olfaction is involved in the earliest interactions between mothers and infants. It helps infants overcome life's first great behavioural challenge, in finding the nipple, plays a key role in the subsequent establishment and maintenance of breastfeeding, and contributes to processes involved in attachment and bonding. Although further research is needed to confirm their effectiveness in clinical practice, we have outlined several ways in which understanding of maternal-infant olfactory communication could inform practical initiatives in breastfeeding initiation and maternal bonding, with corollary benefits for the health and well-being of both mothers and infants.

Further translational research awaits the discovery of the underlying chemical mechanisms. Research is thus required to characterise the volatile organic compounds produced by the lactating breast to which human newborns react, as well as those produced by infants that are reactogenic to mothers. A roadmap for such efforts has been established in the characterisation of the rabbit mammary pheromone, and successful identification could open up novel and practical ways to further facilitate olfactory mediation of breastfeeding initiation, bonding, and responsiveness of primary caretakers.

In the meantime, despite knowledge about the role of odour in nipple-finding, breastfeeding, and bonding that has accumulated over several decades, many clinicians and researchers appear to remain unaware, or at least unconvinced, of its importance, and consideration of olfaction is absent in the most recent WHO/UNICEF BFHI implementation guidance and *Ten Steps*. There is thus a need for greater awareness and enhanced understanding of the critical role of mammary and/or neonatal chemosignals among practitioners, parents, and policymakers alike.

Among practitioners, awareness should be increased at every stage, from hospital policies to training programs for clinical staff. Among parents and caretakers, raising awareness of early olfactory exchanges can begin during antenatal care or breastfeeding counselling and continue long after the return home, when competing or additional socio-cultural odour-related attitudes and practices may come into play. Finally, we advocate that policymakers embed an understanding and awareness of olfactory processes within the BFHI and the *Ten Steps*. By so doing, we can transform an approach to perinatal and postnatal care that currently appears to neglect this fundamental and hidden biological mechanism into one that actively leverages it, not only to promote breastfeeding rates but also to improve bonding, resilience, and the health of mothers and infants worldwide.

## Outstanding questions

The outstanding question we have discussed is: how can we best apply our knowledge of olfactory communication between mothers and newborns to maximise success in breastfeeding and bonding? Important subsidiary questions include: (1) How can such knowledge be optimally incorporated into WHO/UNICEF policy, particularly the BFHI guidance and the Ten Steps? (2) What chemical compounds are responsible for the odour signal that attracts newborns to the breast immediately following birth, and can they be used to facilitate breastfeeding initiation? (3) Do infants produce an olfactory signal of need that influences maternal attentiveness?

**Search strategy and selection criteria**

Literature included in this Personal View was identified by previous research by the authors, supplemented by searches of PubMed, Web of Science, and the references of relevant articles using the search terms “odo∗r”, “olfact∗”, “skin-to-skin”, and “skin to skin”. We referred to papers written in languages other than English where appropriate. Abstracts and meeting reports were included where necessary to support our statements or views.

## Contributors

SCR, BS, and JH conceptualised the paper. SCR and BS wrote the original draft. SCR, BS, FD, KD, JH, DK, BL, AS, PS, VS, and JW acquired funding. FD, KD, JH, DK, BL, AS, PS, VS, JW, TA-M, DB, DD, LJ, SK, LK, ACP, KR, JCS, DS, MS, NW, and ES reviewed the draft paper and gave inputs. SCR, JH, and BS edited the final version. All authors read and approved the final version of the manuscript.

## Declaration of interests

The authors declare that they have no competing interests.
